# Enzootic Gastro-enteritis from Acorns

**Published:** 1898-02

**Authors:** 


ENZOOTIC GASTRO-ENTERITIS FROM ACORNS.—Poncet mentions the appearance of this disease in several cows and bulls. One of the latter relished and freely ate acorns which fell prematurely in consequence of a violent storm, and succumbed to the disease. The others recovered with laxatives, alkalines, and emollient drinks.—*Ann. de Méd. Vét.*

